# Automated interpretation of 3D laserscanned point clouds for plant organ segmentation

**DOI:** 10.1186/s12859-015-0665-2

**Published:** 2015-08-08

**Authors:** Mirwaes Wahabzada, Stefan Paulus, Kristian Kersting, Anne-Katrin Mahlein

**Affiliations:** 1 INRES-Phytomedicine, University of Bonn, Meckenheimer Allee 166a, Bonn, 53115 Germany; 2 IGG-Geodesy, University of Bonn, Nussallee 17, Bonn, 53115 Germany; 3 Computer Science Department, TU Dortmund University, Otto-Hahn-Str. 14, Dortmund, 44227 Germany

**Keywords:** Automatic segmentation, Clustering, 3D-laserscanning, High-throughput, Plant phenotyping

## Abstract

**Background:**

Plant organ segmentation from 3D point clouds is a relevant task for plant phenotyping and plant growth observation. Automated solutions are required to increase the efficiency of recent high-throughput plant phenotyping pipelines. However, plant geometrical properties vary with time, among observation scales and different plant types. The main objective of the present research is to develop a fully automated, fast and reliable data driven approach for plant organ segmentation.

**Results:**

The automated segmentation of plant organs using unsupervised, clustering methods is crucial in cases where the goal is to get fast insights into the data or no labeled data is available or costly to achieve. For this we propose and compare data driven approaches that are easy-to-realize and make the use of standard algorithms possible. Since normalized histograms, acquired from 3D point clouds, can be seen as samples from a probability simplex, we propose to map the data from the simplex space into Euclidean space using Aitchisons log ratio transformation, or into the positive quadrant of the unit sphere using square root transformation. This, in turn, paves the way to a wide range of commonly used analysis techniques that are based on measuring the similarities between data points using Euclidean distance. We investigate the performance of the resulting approaches in the practical context of grouping 3D point clouds and demonstrate empirically that they lead to clustering results with high accuracy for monocotyledonous and dicotyledonous plant species with diverse shoot architecture.

**Conclusion:**

An automated segmentation of 3D point clouds is demonstrated in the present work. Within seconds first insights into plant data can be deviated – even from non-labelled data. This approach is applicable to different plant species with high accuracy. The analysis cascade can be implemented in future high-throughput phenotyping scenarios and will support the evaluation of the performance of different plant genotypes exposed to stress or in different environmental scenarios.

**Electronic supplementary material:**

The online version of this article (doi:10.1186/s12859-015-0665-2) contains supplementary material, which is available to authorized users.

## Background

Recent phenotyping platforms implement a variety of imaging methods, such as 3D-scanning, RGB-imaging, spectral imaging, and/or chlorophyll fluorescence imaging to collect data for quantitative and qualitative studies on plant genotypes in different stress scenarios [1, 2]. The advantage of optical sensor methods in high-throughput screenings is, that a high number of plants can be investigated in time course experiments; and – due to the non-destructive nature of the sensors – the same individual can be observed over time (in contrast to analytical and destructive approaches). Furthermore these sensor methods eliminate the human bias which always occurs when plants are rated visually or manually [3, 4]. Although the current state of the art in sensing plants is far from fully recapitulating entire plant systems, optical sensing systems come close to this ambitious aim. The step towards bridging the ’phenotyping bottleneck’ by technical in plant breeding demands sophisticated sensing approaches and adequate data analysis methods [5–7].

Common methods to assess characteristic and functional parameters of plants from their architecture and geometry by optical sensors are 3D-laserscanning or photogrammetric techniques [8, 9]. Laserscanning has the advantage of a high resolution, combined with a high accuracy, including direct access to the 3D point cloud. These highly resolved 3D point clouds allow an accurate description of the geometry of plant organs and of subtle changes due to abiotic or biotic stress [10]. Plant attributes of relevance which can be deduced from 3D point clouds are plant biomass, growth curves, size and number of relevant plant organs, proportions among single plant organs (i.e. leave, stem and ears of cereals), or shape parameters (product quality).

The segmentation of plant organs is an important task in data analysis. In literature different approaches were proposed. One strategy is the use of a preprocessed mesh representation, and a manual partition of the mesh into morphologic regions [9]. This step has recently been automated [11], but still requires the preprocessed mesh representation of the 3D measurements. Other works aiming at the classification of laser scanned data are used in robotics, e.g. for object or scene recognition/interpretation. For instance, methods that can be subordinated under collective classification approaches take the surrounding information of a point into account. However, they often rely on complex algorithms, are time consuming, and much research has gone into the direction making them more efficient (see [12] and references). One way for identification and segmentation of plant organs without time and labor intensive preprocessing are surface feature histograms. As it has been shown before in Paulus et al. [8], they are an innovative and suitable method for plant organ parametrization from 3D data. These histograms have been developed to recognize geometric primitives in 3D point clouds, where e.g. planes, cylinders and spheres show specific and easy to distinguish histograms. The reason why plants organs lead to specific feature histograms and provide a good separation is that leaf and stem very well correspond to primitives like plane or cylinders, for example. It has been previously shown, that this method is independent to the point to point distance and applicable to multiple plants. Therefore, the surface feature histograms provide an interpretation based on the geometry of the surface and can be used as input for machine learning algorithms like Support Vector Machines (SVM) [13]. As the histogram representation is influenced by the points neighborhood, it makes the application of algorithms such as SVM’s also possible in general. However, for classification a crucial amount of prior knowledge is important. Until now these approaches require a manual supervision of the model after the data is measured. A fully automated data analysis cascade is missing but highly desirable, to save the time and cost for manual labelling the training data by skilled operators.

Triggered by this, we tackle the challenge of how to efficiently analyze this huge amount of data. In particular, we investigated the question *"Can machines help to facilitate the segmentation of plant organs if no labeled data is given?"* and show that this is indeed the case. Specifically, we group the surface feature histograms, acquired from 3D point clouds, using unsupervised clustering approaches. The benefit of unsupervised methods is that they can be used for exploratory data analysis and do not require labeled data, such as class information. A common and widely used method for this is k-means clustering using the Euclidean distance, for which good approaximation guarantees are known. However, since our data consists of normalized histograms, using solely the Euclidean distance may be not appropriate. Consequently, we propose a data driven approximation approach that is based on mapping the data into a different space in a preprocessing step. More precisely, since the histograms can be seen as points on a probability simplex, we propose to map the data from the simplex into Euclidean space using Aitchison geometry [14–16] or into the positive quadrant of the unit sphere [17]. This, in turn, makes it possible to employ the Euclidean distance to measure the similarities between normalized histograms in the space mapped to. Actually, since we change the way we represent the data, any standard methods devised for the Euclidean space can be used. For instance, matrix factorization methods [18, 19] become applicable, where k-means is subordinated. Additionally, based on distance computations we can compute an hierarchical decomposition of the data [20], which can also be used in context of spectral clustering [21]. Furthermore, the proposed approach can also be beneficial for supervised learning, such as SVM’s using RBF-kernel, where a common choice is the squared Euclidean distance.

**Fig. 1:**
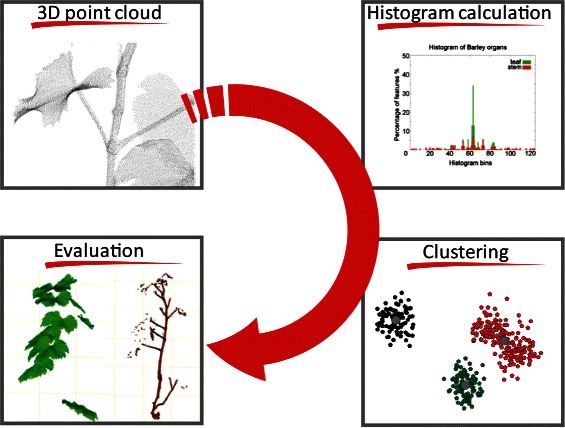
An automated approach for plant organ segmentation of 3D laserscanned point clouds. After data acquisition with a 3D laser scanner, histograms were calculated on the point clouds. These histograms were used for clustering the data by k-means. In a final step the evaluation of the result regarding accuracy, speed and applicability was conducted

Overall, in the present paper we introduce the first fully automated and data driven approach for segmentation and identification of plant organs from 3D point clouds, as summarized in Fig. 1. The developed data mining cascade demonstrates their robustness and applicability on monocotyledonous and dicotyledonous crop plants with diverse shoot architecture.

## Methods

The work flow of the current paper is illustrated in Fig. 1. After data acquisition with a 3D laser scanner, histograms were calculated on the point cloud data. These histograms were used for clustering the data. In a final step the evaluation of the result regarding accuracy, speed and applicability was conducted.

**Notation:** We denote vectors by lower case letters ($\vec {x}$); a real-valued vector of size *m* is written as $\vec {x}\in \mathbb {R}^{m}$; subscripted lower case italic (*x*_*j*_) refer to the components of a vector; matrices are written as bold upper case letters (***X***); a real-valued *m*×*n* matrix is written as $\textit {\textbf {X}}\in \mathbb {R}^{m\times n}$ or using the shorthand *X* ^*m*×*n*^.

### Histogram calculation

Histogram based surface representations have been proven to enable the identification of geometrical primitives in low-resolution point clouds acquired on robotic carrier systems [22]. Coming from robotics, point feature histograms were originally used for the detection of basic geometric shapes in low-resolution laser scans [22, 23] and for a registration of different laser scan viewpoints [24]. Surface feature histograms, a histogram advancement, recently showed their applicability for the segmentation of organs on grapevine and wheat [8], as well as in barley for an organ based parametrization in time course experiments [25]. These histograms encode the information of the surface as e.g. curvature using the neighbourhood of a point and the surface normals. This curvature is characteristic for the surface of e.g. plant leaves and stems and can be used as an input for machine learning methods like SVM to classify these organs automatically. Different geometrical features were calculated and their value domain is subdivided into 5 subregions. Each combination of these subregions corresponds to one histogram bin. By this, a representation of the geometrical neighborhood of one point in the 3D space by a histogram including 125 (histogram) bins is possible.

To calculate the histograms we used the algorithm, as given in [8]. The radius for the normal- (*r*_*N*_) and radius for the histogram-calculation (*r*_*H*_) (where the *r*_*N*_ has to be smaller than the *r*_*H*_) are the two parameters which have to be adapted for every plant type. Before determining the histograms, for each point $\vec {z}_{i}\in \mathbb {R}^{3}$ in the point cloud the normal $\vec {\xi }_{i}$ is computed by considering all point in the range *r*_*N*_. The normal can be determined using the principal component analysis and corresponds to the eigenvector with the smallest eigenvalue. Then, the angular variations between the point $\vec {z}_{i}$ and each point $\vec {z}_{j}$ in the range of *r*_*H*_ are determined using [22, 26]

(1) $$\begin{array}{@{}rcl@{}} \tau_{0} &=& \langle \vec{v},\vec{\xi}_{j} \rangle, \end{array} $$

(2) $$\begin{array}{@{}rcl@{}} \tau_{1} &=& \frac{\langle \vec{u},\vec{z}_{j}-\vec{z}_{i} \rangle}{d(\vec{z}_{j},\vec{z}_{i})}, \end{array} $$

(3) $$\begin{array}{@{}rcl@{}} \tau_{2} &=& \arctan\left(\langle \vec{w},\vec{\xi}_{j}\rangle,\langle \vec{u},\vec{\xi}_{j}\rangle\right), \end{array} $$

where $\vec {u}=\vec {\xi }_{i}$, $\vec {v} = (\vec {z}_{j}-\vec {z}_{i})\times \vec {u}$, $\vec {w} = \vec {u}\times \vec {v}$, $\langle \vec {x}, \vec {y} \rangle $ is the scalar product and $d(\vec {x},\vec {y})$ denote the Euclidean distance between the points (see next section). Given the features one can build single point histograms $\vec {x}_{sp_{i}}$, where the index *idx* of the histogram bin in which the points $\vec {z}_{i}$ and $\vec {z}_{j}$ falls is computed using

(4) $$\begin{array}{@{}rcl@{}} idx = {\sum\nolimits_{s=0}^{2}} \left[\frac{\tau_{s} b}{\tau_{s_{\max}}-\tau_{s_{\min}}}\right]b^{s}. \end{array} $$

Here, *b* represents a division factor defining the size of the histogram. Then, to better capture the complex structures, such as stems or leaves, we build weighted histograms $\vec {x}_{\beta }$ out of the neighbors single point histograms $\vec {x}_{\textit {sp}}$ in the range *r*_*H*_ for the point $\vec {z}_{i}$ using

(5) $$\begin{array}{@{}rcl@{}} \vec{x}_{\beta_{i}} = \sum\limits_{d\left(\vec{z}_{j},\vec{z}_{i}\right)\le r_{H}} \beta_{j}\vec{x}_{sp_{j}}+(1-\beta_{j})\vec{x}_{sp_{i}}, \end{array} $$

where *β* is a weight function $\beta _{j}= 1-\left (0,5+\frac {d(\vec {z}_{i},\vec {z}_{j})}{r_{H}}0.5\right)$. The use of the weights *β* for the calculation of the final histograms ensures that histograms of points near the limit of the radius *r*_*H*_ have lower impact than those closer tho the point $\vec {z}_{i}$. For a detailed description we refer to Paulus et al. [8].

### Metrics for measuring histogram similarity

A major part of the present work consists of providing metrics for comparison of histograms obtained from 3D laser point clouds, and using them for unsupervised learning for automated classification or clustering of plant organs. A common and widely used measure is the Euclidean distance, which is defined as

(6) $$\begin{array}{@{}rcl@{}} d(\vec{x},\vec{y}) = \sqrt{{\sum\limits_{i}^{m}}(x_{i}-y_{i})^{2}}, \end{array} $$

for two vectors $\vec {x},\ \vec {y} \in \mathbb {R}^{m}$. For instance, for clustering objects one can use the k-means algorithm, where the task is to minimize the squared Euclidean distance of data points to its nearest cluster representatives (see [27] for a description), for which good approximations guarantees are known. Thus, for a given dataset containing *n* observations $\textbf {X} = \{\vec {x}_{1},\dots,\vec {x}_{n}\}$ with $\vec {x}_{i}\in \mathbb {R}^{m}$ the goal in k-means is to minimize

(7) $$\begin{array}{@{}rcl@{}} E={\sum\limits_{i}^{n}}{\sum\limits_{j}^{k}}\zeta_{ij}d(\vec{x}_{i},\vec{\mu}_{j})^{2}. \end{array} $$

Here, $\vec {\mu }_{j}$ denotes the cluster representative, *ζ*_*ij*_ is binary, that is *ζ*_*ij*_∈{0,1}, describing the cluster membership of a data point *x*_*i*_ to cluster *j*.

However, using the Euclidean distance directly for analyzing surface feature histograms is not a sensible idea, as it is known to be sensitive to noise and does not generalize well [28]. Therefore, we propose a data driven approach by looking at the properties of the data itself. Since the histograms represent proportions that sum to one, they can be considered to be samples from a probability simplex. In other words, we are interested in clustering normalized histograms on the simplex. For doing this, we consider two different approaches that are based on simple data transformation as preprocessing. The presented approaches are not only easy-to-realize but still employ the Euclidean distance for measuring histogram similarities. In turn standard algorithms for clustering or classification of normalized data, for example, can be used.

In the following we will focus on k-means, as it is a simple and widely used method for clustering objects and a number of efficient implementations exists for parallel and streaming settings [29]. Since we use it here for clustering normalized histograms, we will discuss and motivate two approaches for measuring the histogram similarity.

#### Hellinger distance

To arrive at an automated clustering approach for histograms, we propose to transform the data before computing similarites/differences between feature point histograms. For instance, it has been shown that using Kullbalk-Leibler (KL) divergence can achieve superior results when measuring the similarity between histograms [28]. To get a clustering with respect to KL-divergence one may use an approximation based on the Hellinger distance, which was also shown to be more sensitive to the differences in smaller bins [30]. The Hellinger distance for two histograms $\vec {x}$ and $\vec {y}$ is given by

(8) $$\begin{array}{@{}rcl@{}} d_{H}(\vec{x},\vec{y}) = {\sum\limits_{i}^{m}}\left(\sqrt{x_{i}}-\sqrt{y_{i}}\right)^{2}. \end{array} $$

This, in turn, is equivalent to the square of the Euclidean distance, as given by Eq. (6), between the square root of two data points $\vec {x}$ and $\vec {y}$. Thus, clustering of data using square root transformations and k-means should lead to a good clustering in terms of minimizing Hellinger distance between each object and its nearest cluster center. It can be shown that this yields an *O*(log*n*) approximation of clustering based on minimizing KL-divergence [17]. However, KL-divergence do not satisfy the metric properties, i.e. it is is not symmetric and do not satisfy the triangle inequality. The latter point holds also for its symmetric alternatives, such as Jeffrey’s Divergence [31].

To cluster with respect to Hellinger distance, we therefore consider a data driven procedure. Our strategy is to apply square root transformation (*SQr*) before clustering. That is, we set

(9) $$\begin{array}{@{}rcl@{}} \vec{y} = SQr(\vec{x})=\sqrt{\vec{x}} = \left(\sqrt{x_{1}},\ldots, \sqrt{x_{m}}\right), \end{array} $$

i.e. transform the data from simplex space into positive quadrant of the unit sphere [17]. The resulting representation, in turn, can be used to find a clustering of histograms, as considered in the paper, using standard implementations of k-means. Since the cluster centers for the mapped data do not lie on the unit sphere, we recompute them using the original histograms and cluster assignments. This make sure that the cluster centers lie on the simplex.

#### Aitchison distance

As an alternative we can follow [14] using the so called log ratio transformations. Here, the idea is to map the data from the probability simplex onto Euclidean space, which makes statistical analysis applicable to the transformed data. For instance, additive log ratio can be used for the modeling, but has some drawbacks if using it to measure the difference between two proportions [16]. To measure differences between two histograms one can use the Aitchison distance [15, 16], which can be written as

(10) $$\begin{array}{@{}rcl@{}} d_{A}(\vec{x},\vec{y}) = \sqrt{{\sum\limits_{i}^{m}}\left(\ln\frac{x_{i}}{g(\vec{x})}-\ln\frac{y_{i}}{g(\vec{y})}\right)^{2}}, \end{array} $$

where $g(\vec {x}) =({\prod ^{m}_{i}} x_{i})^{1/m}= \sqrt [m]{x_{1}\cdots x_{m}}$ denote the geometric mean. It can be easily seen that Eq. 10 is equivalent to Euclidean distance on the transformed data using centered log-ratio (*clr*) transformation, which is given by

(11) $$\begin{array}{@{}rcl@{}} \vec{y} = clr(\vec{x}) = \left(\ln({x_{1}}/{g(\vec{x})}),\ldots, \ln({x_{m}}/{g(\vec{x})}) \right), \end{array} $$

and its inverse $clr^{-1}(\vec {y}) = \left ({\exp {(y_{1})}}/{\sum _{j}\exp {(y_{j})}},\ldots,\right.\left. {\exp {(y_{m})}}/{\sum _{j}\exp {(y_{j})}}\right)$. Thus, we can use *clr* transformed histograms with Euclidean distance within k-means clustering. Note, other transformations, such as isometric logratio transformation [32], may be used as well. It solves the *clr* problem that leads to singular covariance matrix, by preserving its properties like isometry between the simplex and the real space.

However, since the histograms, considered in this work, also consist of empty or zero bins, hence, this leads to numerical problems when computing $clr(\vec {x})$ due to the logarithm and as also the geometric mean in the denominator is $g(\vec {x})=0$ if any *x*_*j*_=0 for *j*=1,…,*m*. Finding a good choice for replacing them is essential when using log ratio transformations (see [33] and references), e.g. for missing or rounded values. For the histogram analysis, Wahl et al. [28] suggested to replace the zero bins by a small common value, which is lower as the smallest non-zero value. For the experiments in the current work we used a simple procedure by adding a small value *ε* to all data points. It has shown that using this approach will lead to a better clustering using *clr* approach, compared to replacing only zero bins across different datasets. Note, by contrast, for the *SQr*-approach we do not need to care about the zero bins.

### Histogram clustering algorithm

The overall procedure for clustering the normalized histograms acquired from 3D point clouds is summarized in Algorithm 1. We start by transforming the data using either *SQr* or the *clr* approach [lines 1–4]. Then, on the new representation of the data, we run k-means clustering in [lines 5–14], which can be done using an EM-algorithm by iteratively optimizing the cluster memberships which are stored in a matrix *Z* [lines 8–10] (E-step) and computing the cluster representatives in matrix *M* [lines 11–13] (M-step). Finally we determine the cluster representatives on the simplex $\pmb {\tilde M}$ using the inverse centered log ratio transformation for the *clr* approach. For the *SQr* approach we use the cluster assignments in *Z* and the original inputs *X* to get the final cluster centers.

However, as we transform our data before clustering and do not change the underlying algorithms, the time complexity remains the same. For the transformations we need only one pass over the entire dataset. This, in turn, can be easily parallelized or can also be done sequentially, to overcome memory issues. Using k-means as given by Algorithm 1 [lines 5–14] enables to find a local optimum, whereas finding of a global optimum is an NP-hard problem [34], even for k = 2.

### Data acquisition

The data was acquired with the 3D measuring combination of an articulated measuring arm (Romer Infinite 2.0 (1.4 m), Hexagon Metrology Services Ltd., London UK) and laser triangulation sensor (Perceptron Scan Works V5, Perceptron Inc., Plymouth, MI, USA). This combination has been proven regarding applicability for plant measuring and accuracy for the scanning of grapevine, wheat and barley [8, 10]. It provides an accuracy of about 45 *μ* *m* for points within the 2D-scanning field. The single 2D-scan lines were combined automatically by the articulated measuring arm to a 3D point cloud. The measuring arm enables imaging an almost occlusion free point cloud by using many different points of view. The point cloud was processed using Geomagic Studio 12 (Raindrop Geomagic Inc, Morrisville, NC, USA).

The preprocessing of the point cloud is limited to the cutting of scanned objects that do not belong to the focussed object. Furthermore the point cloud density is reduced to an uniform grid of 0.5 *m* *m* point to point distance, this is necessary due to the scanning method that produces an inhomogeneous point resolution all over the point cloud according to the speed that sensor is moved over the object.

### Datasets

In our experiments we used different datasets of plants including grapevine, wheat, and barley, as shown in Additional file 1. Each dataset was processed as explained above to get a histogram representation:

- **Grapevine** (stem, leaves): The grapevine plants (*Vitis vinifera ssp. vinifera, variety Mueller Thurgau*) were grown in commercial substrate in plastic pots ($\varnothing 170~mm$) under greenhouse conditions. The plants were watered and fertilized on demand. Environmental parameters were kept constant at 23/20 °C (day/night), 60 % relative humidity and a photoperiod of 16 h. The measurement was done at growth stage 19 (according to BBCH, [35]). We had a total number of *n*=55635 calculated histograms, each with a length of *m*=125. For our evaluation we could make use of label information (stem and leaf), which were set manually by a human annotator.
- **Grapevine** (berry, rachis): The second grapevine datasets (*Vitis vinifera ssp. vinifera, variety Mueller Thurgau*) included the berries and the rachis. It was grown on a vineyard at Geilweilerhof, Sindelfingen, Germany in Summer of 2012. This point cloud consisted of a total number of *n*=57989 histograms. For this dataset no label information was given, because the segmentation is even manually very hard.
- **Wheat**: The wheat plants (*Triticum aestivum, variety Taifun*) were grown in plastic pots ($\varnothing 200~mm$) under similar conditions as the grapevine plant. The measurement was done at growth stage BBCH 85. The dataset consisted of *n*=215090 histograms. For this dataset manually determined labels for histograms on the ear, stem and leaves were provided.
- **Barley**: Additionally we used three barley datasets (*Hordeum vulgare L*, CV. Barke). They were grown in plastic pots ($\varnothing 16~cm$) in a green house under similar conditions as the grapevine plant. The measurements followed the same plant at different developing stages (19, 26, 31 days after sowing). They consisted of a total number of *n*=15064 (plant 1, BBCH 12), *n*=41167 (plant 2, BBCH 21) and *n*=139465 (plant 3, BBCH 23) histograms. For each histogram the labels (leaf or stem) were provided and used for the evaluation.

All histogram calculations used fixed radii for the normal- and histogram calculation *r*_*N*_=2.5 and *r*_*H*_=12.5 according to [8].

## Results and discussion

The main goal was the comparison of data-driven approaches for clustering feature histograms of grapevine, wheat and barley plants using the following settings:

- **KM:** histogram clustering using using k-means and Euclidean distance on normalized histograms directly.
- **HC-1:** histogram clustering where we transformed the data using Eq. (9) before processing.
- **HC-2:** histogram clustering, where the data was transformed using *clr* approach as given by Eq. (11), before processing.

In this work we used a simple procedure for replacing the zero bins by adding a small value $\epsilon =\frac {1}{m}$ to all data points, where *m* denotes the number of bins used for histogram computation, and normalized the data before computing the *clr* transformation. This led to similar or better clusterings compared to other settings in the range 10^−16^≤*ε*≤10^−1^. Note, the zero bins were replaced only for computing the HC-2, whereas for HC-1 we used the original inputs directly.

With respect to application within plant phenotyping, the needed amount of clusters is often known or given before/or during the experiment, as one is looking for specific plant organs. As long as it is aimed to separate leaves and stems, it is recommended to use two clusters, one for each organ. Using more clusters enables the recognition of further classes like inlaying berries or leaf border points which have not been focused before. However, in such cases determining the number of clusters automatically may be crucial; we left this questions for the further work. For the sake of better visualization we show for the qualitative results in the following only clusterings learned for a small number of clusters. All experiments were conducted on a standard computer with 3.2 GHz Intel Core i7-3930K and 16 GB main memory.

### Quantitative comparison of histogram clustering approaches

For a quantitative comparison we used the results of an automated segmentation with labels. The labels themself were the outcomes of a manual annotation by a human annotator. For evaluating the clustering, we consider two commonly used measures. First we consider *F-measure*, which can be seen as the harmonic mean of the precision and recall that are known from information retrieval [36, 37]. It can be computed for a clustering as follows

(12) $$  F = \sum\limits_{i}\frac{n_{i}}{n}\max{F(i,j)}~~\text{with}~~ F(i,j)=2\frac{P(i,j) R(i,j)}{P(i,j)+R(i,j)},  $$

where *n*_*i*_ is the number of histograms with a particular label *i*, *R*(*i*,*j*) denotes the recall and *P*(*i*,*j*) the precision of a class *i* for a cluster *j*. A good clustering should have a higher *F-measure* value. However, the *F-measure* does penalize also the number of clusters, since each class is judged by the cluster with the highest number of histograms with that label. In order to consider the distributions of labels within each cluster we additionally use the *entropy* measure [38]. It can be determined using

(13) $$ entropy = \sum\limits_{j} \frac{n_{j}}{n} E(j) ~~\text{with} ~~E(j) = -\sum\limits_{i} \frac{n_{ij}}{n_{j}}\ln\frac{n_{ij}}{n_{j}},  $$

where *n*_*ij*_ denote the number of histograms with label *i* in cluster *j* and *n*_*j*_ the total number of objects in cluster *j*. A lower *entropy* value stands for a better clustering, indicating that clusters contain mostly objects with similar labels.

**Fig. 2:**
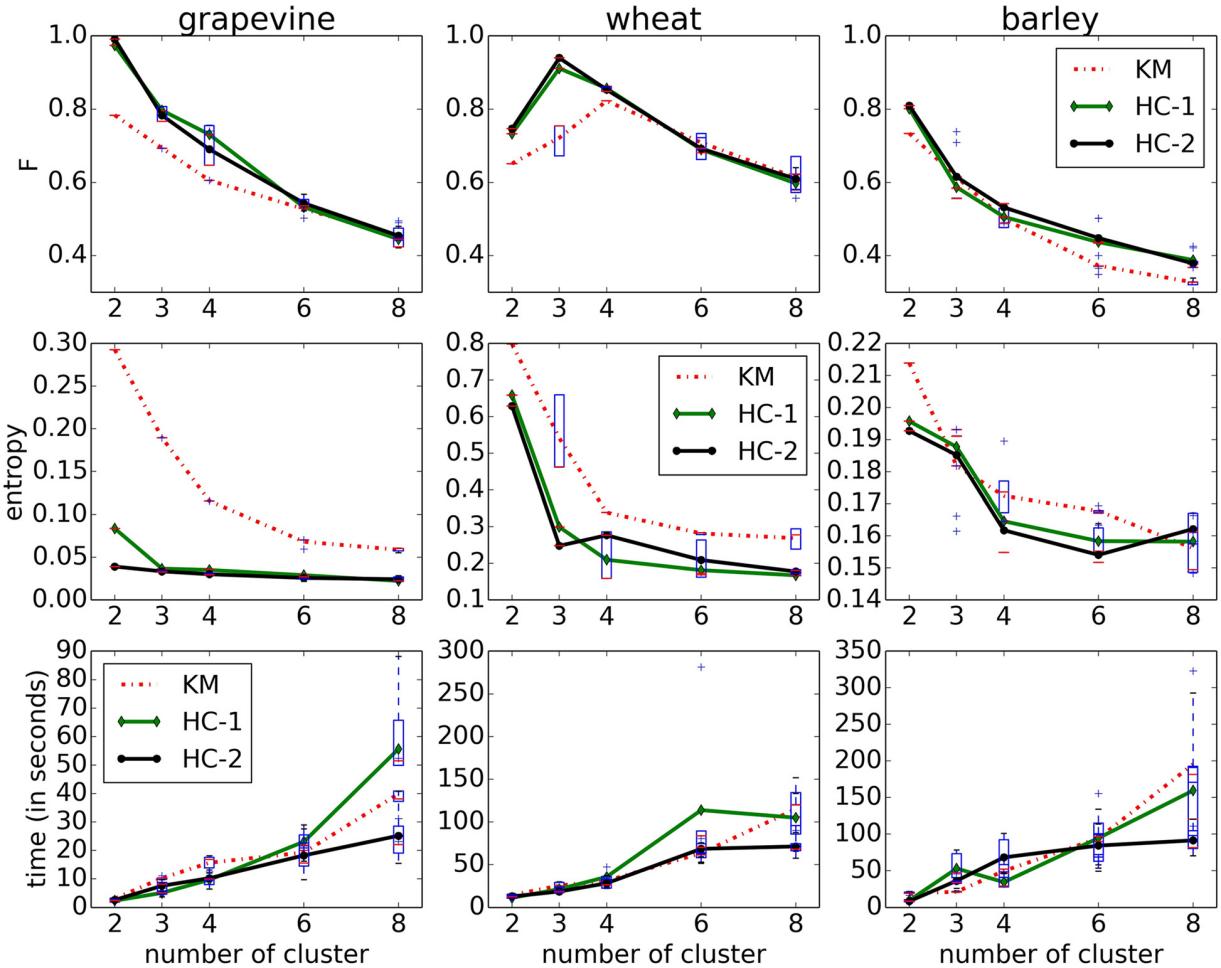
Quantative results showing the *F-measure* and *entropy* values as a function of number of clusters. The *F-measure* results (top row) show a better performance for Algorithm 1 using data mappings (HC) than those for k-means (KM) clustering on normalized histograms directly. This is also captured by the entropy values (middle row), as it considers the distributions of different labels within the clusters. The lower value, the more the clusters are dominated by histograms of a particular label, and therefore the better the clustering. For all methods the algorithm required only few minutes per run and the number of cluster (bottom row)

Figure 2 summarizes the results for the grapevine, wheat and barley datasets where manual annotations were given. For the grapevine dataset consisting of stem and leaves, as well as the wheat dataset with leaves, stem and ears, we computed separately the clusters, whereas for clustering the barley data containing of leaves and stems, we considered the histograms of all three datasets together. Since k-means is based on random initialization, which consequently can lead to different clusterings, each experiments was repeated five times to provide reliable results. We report averaged values of all runs as a function of number of clusters.

The *F-measure* in Fig. 2 (top row) clearly show that histogram clustering using data transformations outperforms the naive method on all datasets. The best results are achieved if the number of clusters is equal to the number of different labels, which is *k*=2 for grapevine and barley dataset, and *k*=3 for wheat dataset. Additionally, the middle row in Fig. 2 shows the *entropy* results. A lower value indicates that the clusters contain mostly histograms with a particular label. Here, using histogram clustering, as given by Algorithm 1, outperforms the direct application of k-means clustering for grapevine and wheat dataset. For the barley data set it is comparable or better than k-means. The lower value for the larger number of classes indicates a better separation between leaves and stems for all methods. For grapevine and wheat dataset the differences are small, which indicates that we are already good even for lower number of cluster. For all datasets the algorithm required only few minutes per run and number of clusters (*k*=2,…,8) to get the clustering, as shown in Fig. 2 (bottom row).

### Automated identification of plant organs

**Fig. 3:**
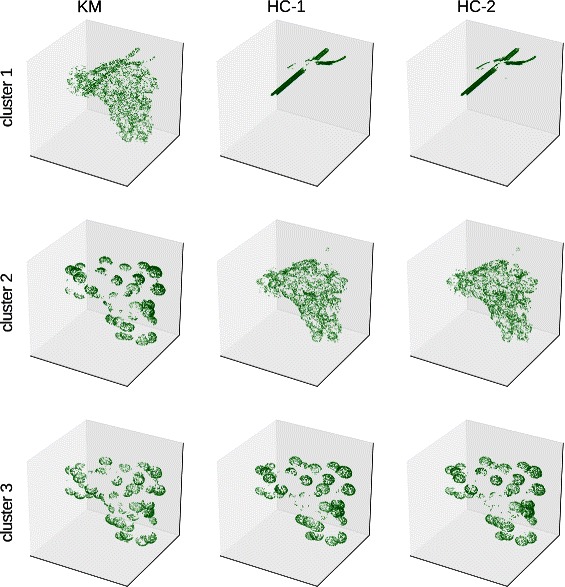
Example for clusterings of the grapevine dataset (berry and rachis) using Algorithm 1 and with different data mappings (*k*=3). For each cluster a subset of points are illustrated, containing the most histograms located on the rachis and parts containing the berry surface and the inner parts of the fruit

**Fig. 4:**
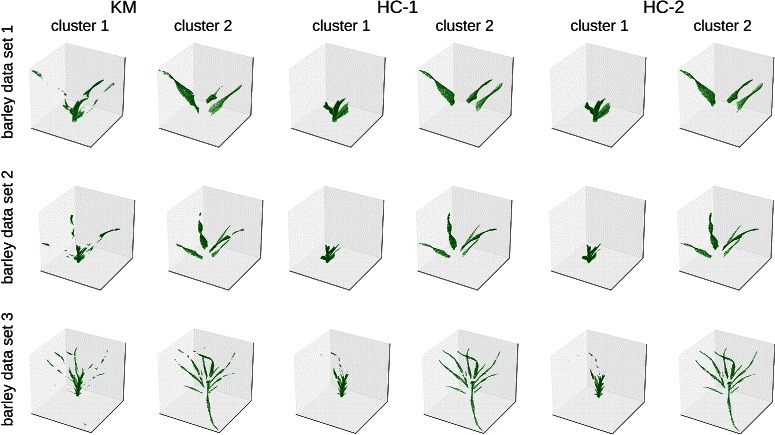
Example for clusterings the three dataset consisting of a barley plant at different developing stages (BBCH 12, 21 and 23) using Algorithm 1 with different data transformations (*k*=2). For each cluster a subset of points are illustrated, containing the most histograms located on the stems (cluster 1) and leaves (cluster 2)

In addition to the quantitative analysis, we report qualitative results achieved from all datasets. For that we additionally consider the clustering on the second grapevine dataset consisting of berry and rachis for which no manual annotations were available. The clusters achieved by using all three methods are illustrated in Fig. 3 for the grapevine dataset and in Fig. 4 for the barley dataset.

In the results for the grapevine dataset it was possible to distinguish between rachis and parts containing the berry using histogram clustering approaches HC-1 and HC-2 (Fig. 3). More interestingly, the clusterings can distinguish between the berry surface, where individual grapes are well captured by the 3D laserscans, and parts belonging to the inner parts of the fruit. However, using k-means directly does not capture this well, as shown in Fig. 3 first column. It needed one more cluster (*k*=4) to separate berry and rachis parts, but also required one more cluster to describe the parts on the fruit, compared to other methods. Interestingly, the clusters achieved for the barley dataset show a more accurate differentiation of different parts and are more coherent if using Algorithm 1 and data mappings (HC), compared to running k-means (KM) directly. This is illustrated in Fig. 4 first column, where also big parts on the leaves are assigned to the cluster containing the histograms from stem. By contrast, using HC-(1,2) lead to more clearly distinguished clusters, that also can facilitate further labeling of the data. However, in cases when very large datasets and varying dimensionalities need to be analyzed, finding a good choice for *ε* to replace zero bins can be time consuming and tricky if using HC-2 (*clr* approach). Therefore, the use of HC-1 (*SQr* approach) may be an option, as it also led to results of similar quality compared to those found by HC-2. The results for the remaining datasets are shown in Additional files 2 and 3 and can be thought of as another justification of quantitative results, discussed in the previous subsection.

**Fig. 5:**
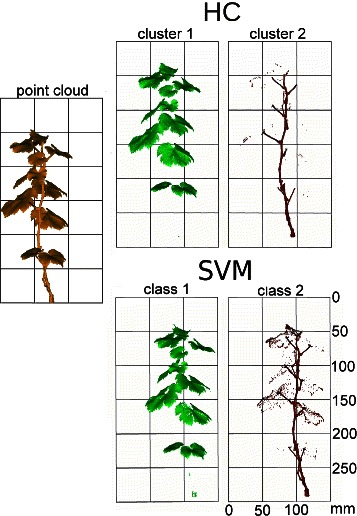
Automated clustering of a grapevine point cloud using Algorithm 1 provides a clear separation of the plant organs leaf and stem. Minor misclassified regions appear at the transition between the organs and at leaf edge points. For the comparison we show the SVM classification results of the same dataset as determined in the study presented in [8]

Additionally, we qualitatively compared the results of HC-2 to classification using SVM (as presented in [8]) for the grapevine dataset consisting of stem and leaves. The results are shown in Fig. 5. Minor misclassified regions appeared at the transition between the organs and at leaf edge points using both methods. The k-means results were computed without using any label information, whereas for the classification using SVM training data was required. It was provided through manual, time consuming labeling. However, the classification task is of great importance for organ differentiation, here we could make use of histogram transformation before learning the classifier or additionally incorporate clustering into active learning [39]. This, in turn, will lower the manual efforts in cases where no training data, which are required in supervised settings, is available. We left this question for the further work.

In general, the results show that the time consuming and costly work of manual labelling can be automated in high precision. Furthermore, the clustering with an undefined amount of clusters for regions of points with similar surface structure become visible. This helps to get a deeper knowledge of the plants/organs structure as it is now possible e.g. to access transition regions between single organs. Moreover, by using unlabeled data we could show that our clustering enables an organ segmentation even when manual labelling is very hard or almost impossible. Interestingly, the clustering of the grapevine fruit enabled the segmentation of the inner skeleton which is hard to access by the human eye.

## Conclusions

Modern plant phenotyping with diverse sensors and exhaustive time series measurements of multiple replicates arose an increasing demand for task orientated data analysis solutions. The present paper provided data driven approaches for plant organ segmentation that make the use of standard algorithms, such as k-means with the Euclidean distance, possible. Actually any data analysis method that build on similarities or distance computations between surface feature histograms, acquired from 3D point clouds, is applicable. We achieved an automation of the data analysis pipeline and a reduction of prior knowledge for the interpretation of plant surfaces. By clustering the histogram representation, different classes of the input point cloud could be identified and separated. Our approach shows that manual labeling can be automated. This approach can especially be used when manual labeling becomes extremely hard due to occlusion or in case that is only possible by viewing from a specific direction. Automated labeling allows the segmentation of un-intuitive surface regions, which enables a more objective way for surface segmentation of plants. Besides getting fast insights on the data one may additionally use the result of automated clustering to subsequently support active learning approaches. Current state-of-the-art research in developing descriptors for 3D surfaces [40] suggests that our method can easily be transferred to various 3D descriptors like Spin Images, Shape Context or Local Surface Patches. The presented data analysis pipeline will speed up the assessment of geometrical features in high-throughput plant phenotyping.

## Additional files

Additional file 1
**3D point clouds for the datasets considered in this work.** We used data from grapevine point clouds (top row, left), consisting of leaf and stem at growth stage BBCH 19, and a second grapevine dataset including the berry and its rachis. Additionally we measured wheat plants (top row, right) at grown stage BBCH 85 and point clouds of a barley plant (bottom row) taken at different developing stages (BBHC 12, 21 and 23).

Additional file 2
**Clusterings for the grapevine dataset consisting of stem and leaves.** Example for clusterings of a grapevine using Algorithm 1 with different data transformations (*k*=2). For each cluster a subset of points are illustrated, containing the most histograms located on the leaves (top row) and stem (bottom row).

Additional file 3
**Clusterings for wheat dataset consisting of leaves, stems and ears.** Example for clusterings of a dataset consisting of wheat using Algorithm 1 with different data transformations (*k*=3). For each cluster a subset of points are illustrated, containing the most histograms located on the leaves (top row), ears (middle row) and stems (bottom row).
