# Supplementary figures and images for: Automated interpretation of 3D laserscanned point clouds for plant organ segmentation
Source: BMC Bioinformatics. 2015 Aug 8;16:248. doi: 10.1186/s12859-015-0665-2 (PMC4528849; doi:10.1186/s12859-015-0665-2)

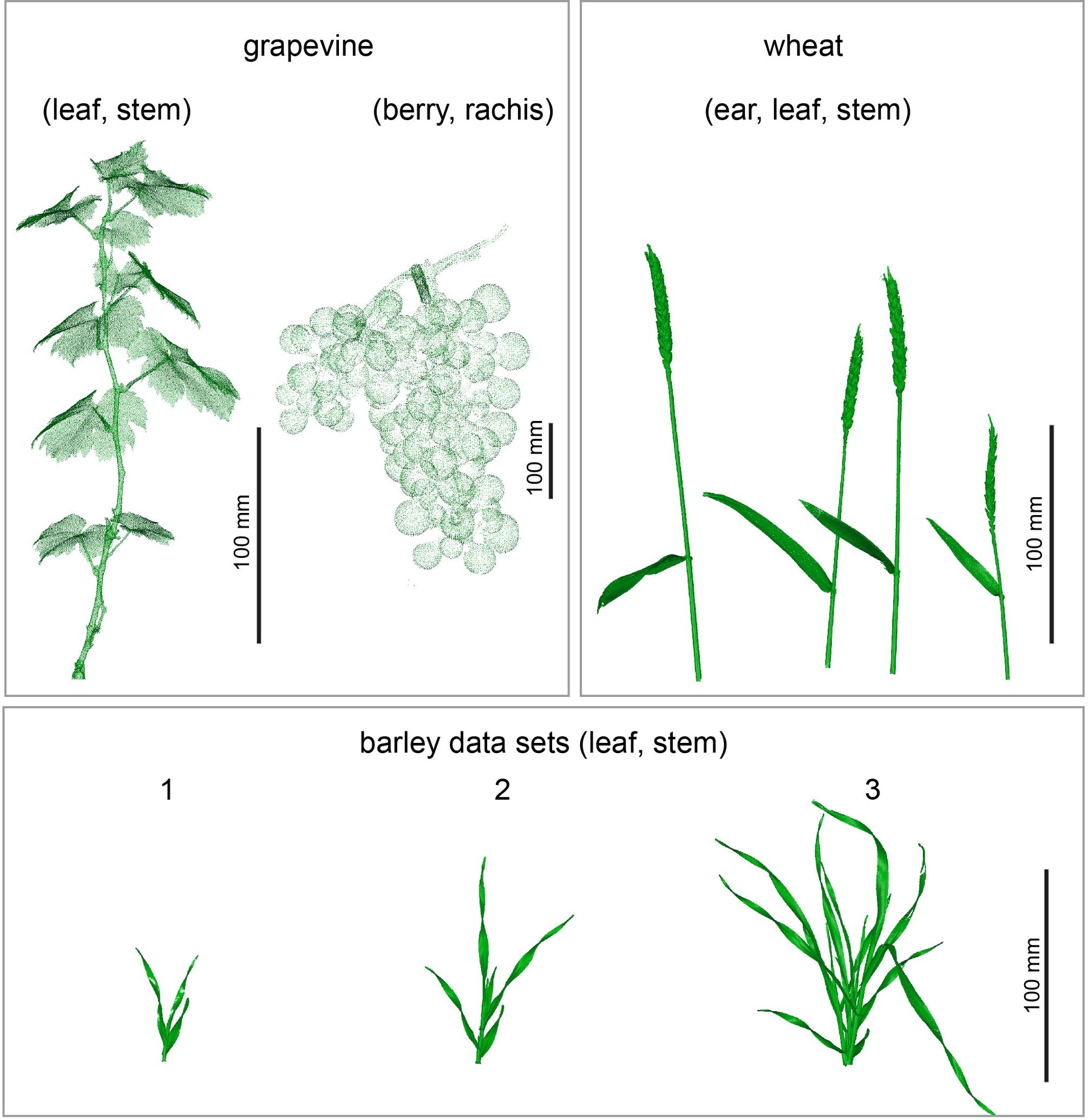

Supplement: Additional file 1 — 3D point clouds for the datasets considered in this work. We used data from grapevine point clouds (top row, left), consisting of leaf and stem at growth stage BBCH 19, and a second grapevine dataset including the berry and its rachis. Additionally we measured wheat plants (top row, right) at grown stage BBCH 85 and point clouds of a barley plant (bottom row) taken at different developing stages (BBHC 12, 21 and 23). [file 12859_2015_665_MOESM1_ESM.jpg]

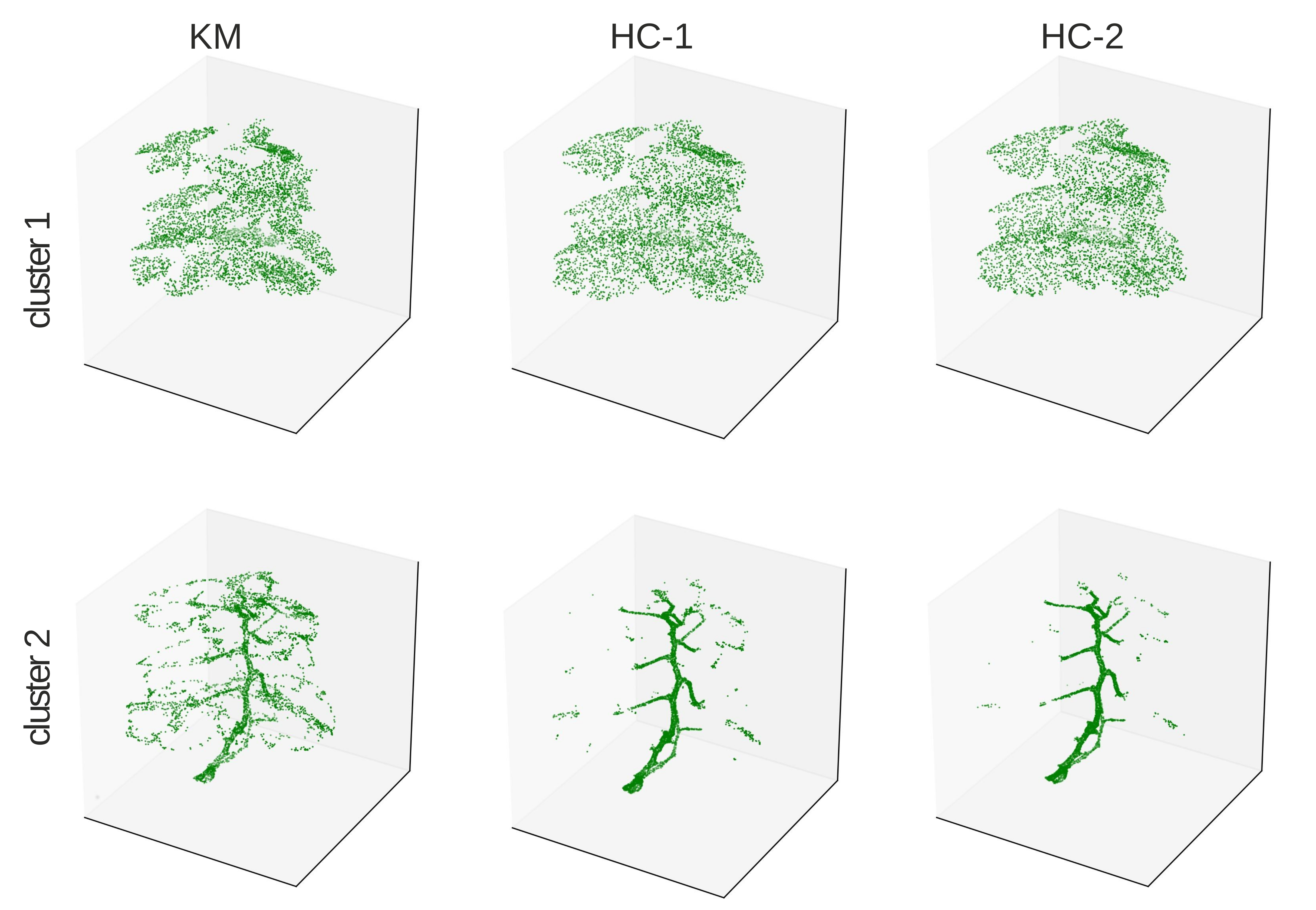

Supplement: Additional file 2 — Clusterings for the grapevine dataset consisting of stem and leaves. Example for clusterings of a grapevine using Algorithm 1 with different data transformations (k=2). For each cluster a subset of points are illustrated, containing the most histograms located on the leaves (top row) and stem (bottom row). [file 12859_2015_665_MOESM2_ESM.jpg]

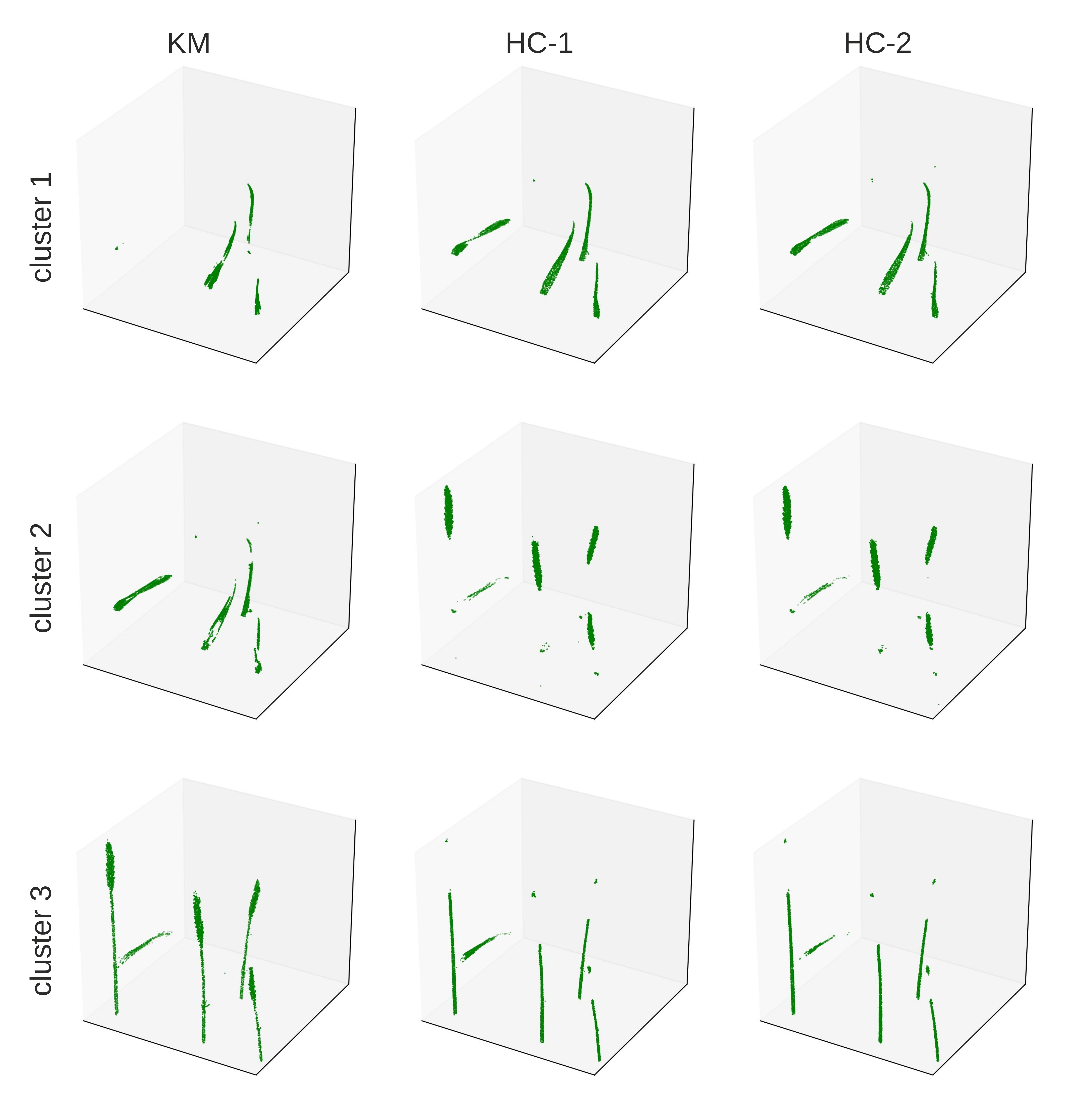

Supplement: Additional file 3 — Clusterings for wheat dataset consisting of leaves, stems and ears. Example for clusterings of a dataset consisting of wheat using Algorithm 1 with different data transformations (k=3). For each cluster a subset of points are illustrated, containing the most histograms located on the leaves (top row), ears (middle row) and stems (bottom row). [file 12859_2015_665_MOESM3_ESM.jpg]
